# Endothelial Cells and Astrocytes: A *Concerto en Duo* in Ischemic Pathophysiology

**DOI:** 10.1155/2012/176287

**Published:** 2012-06-24

**Authors:** Vincent Berezowski, Andrew M. Fukuda, Roméo Cecchelli, Jérôme Badaut

**Affiliations:** ^1^Université Lille Nord de France, 59000 Lille, France; ^2^UArtois, LBHE, EA 2465, 62300 Lens, France; ^3^IMPRT-IFR114, 59000 Lille, France; ^4^Departments of Physiology, Loma Linda University School of Medicine, Loma Linda, CA 92354, USA; ^5^Departments of Pediatrics, Loma Linda University School of Medicine, Loma Linda, CA 92354, USA

## Abstract

The neurovascular/gliovascular unit has recently gained increased attention in cerebral ischemic research, especially regarding the cellular and molecular changes that occur in astrocytes and endothelial cells. In this paper we summarize the recent knowledge of these changes in association with edema formation, interactions with the basal lamina, and blood-brain barrier dysfunctions. We also review the involvement of astrocytes and endothelial cells with recombinant tissue plasminogen activator, which is the only FDA-approved thrombolytic drug after stroke. However, it has a narrow therapeutic time window and serious clinical side effects. Lastly, we provide alternative therapeutic targets for future ischemia drug developments such as peroxisome proliferator- activated receptors and inhibitors of the c-Jun N-terminal kinase pathway. Targeting the neurovascular unit to protect the blood-brain barrier instead of a classical neuron-centric approach in the development of neuroprotective drugs may result in improved clinical outcomes after stroke.

## 1. Introduction: Current Clinical ****Overview of Stroke 

In the United States, stroke is the number one cause of chronic disability and the fourth leading cause of death, with approximately 7 million adults affected [1]. Annually there are approximately 800,000 strokes in the US, of which 87% are ischemic strokes, 10% are primary hemorrhages, and 3% are subarachnoid hemorrhages [1]. Together they cause the country a financial burden of approximately 62.7 billion dollars [2]. Cerebral ischemic stroke is caused by an occlusion of a cerebral blood vessel, typically by a thrombus, which causes a decrease in cerebral blood flow and thus limits the supply of oxygen and nutrients globally (in global ischemia) or to certain regions of the brain (in focal brain ischemia). This absence of blood flow in a brain region causes neuronal death in addition to damaging the vascular tree; the vascular tree is usually made more fragile during the ischemic period and damaged during reperfusion. Time is an important parameter in the evolution of brain injury. In 2006, Saver et al. have estimated the impact of stroke on the brain tissue [3] to be immense; the brain may lose up to 120 million neurons, 830 billion synapses and 714 km of myelinated fibers for each hour after stroke onset [3]. Ischemic stroke seems to accelerate aging of the brain at a rate of 3.6 years each time when the symptoms are not treated [3]. Therefore, the clinical goal of acute stroke treatment is to reduce brain damage by limiting the time of ischemia through thrombectomy (mechanical endovascular approach) or thrombolytic therapy, which consists of in lysing the blood clot in order to restore cerebral blood flow. 

Recombinant tissue plasminogen activator (rtPA) is currently the only thrombolytic molecule administered during acute cerebral infarction that provides a clinical benefit in terms of survival and neurological outcome [4]. The rtPA administration must be within the first 4 hours 30 minutes after stroke onset to maintain the beneficial effects without substantially raising the side effects/risk [5, 6], which limits its use. Based on the organization of emergency care, only 5% of stroke patients are eligible for this therapy in this narrow time window, which leaves the remaining 95% of patients without any beneficial treatment available. The major risk of rtPA is the extension of the damage due to potential bleeding [7]. The need for drug development to prevent the neuronal loss has driven research on neuroprotective agents that aim to save viable neurons located in the ischemic penumbra area. However, all of the proposed neuroprotective treatments specifically targeting neurons that showed promise on the bench have failed in clinical trials [8]. 

In 2000, the neurovascular unit (NVU) was proposed as a physiological unit composed by neurons, astrocytes, and endothelial cells [9]; there is a growing interest in studying the changes of the NVU after stroke. In addition to cell death, ischemic stroke is characterized by changes in the properties of the blood-brain barrier (BBB) with physical disruption of the tight junctions contributing to aggravation of cerebral edema and consequently neuronal death. The new strategy for drug development is to have molecules with a broader spectrum targeting not just the neurons but the NVU as a whole entity. In the present paper, we will focus on some molecular and cellular mechanisms of astrocytes and endothelial cells. We will look specifically at: (1) the ways astrocytes and endothelial cells work in concert in stroke pathophysiology such as BBB disruption and edema formation, (2) how they could be affected after rtPA treatment, and (3) new drug developments in the future.

## 2. Definition of the Neurovascular/Gliovascular Unit

Several groups have proposed the NVU as a physiological unit composed of not only endothelial cells, astrocytes, and neurons but also pericytes, smooth muscle cells, and the interacting circulating peripheral immune cells [10–12]. The term “gliovascular” emphasizes the importance of the interactions between astrocytes and cerebral blood vessels within the NVU [13], which are critical in cerebral blood flow regulation [14], brain energy metabolism [15], and also the maintenance of the BBB properties [13]. 

**Figure 1:**
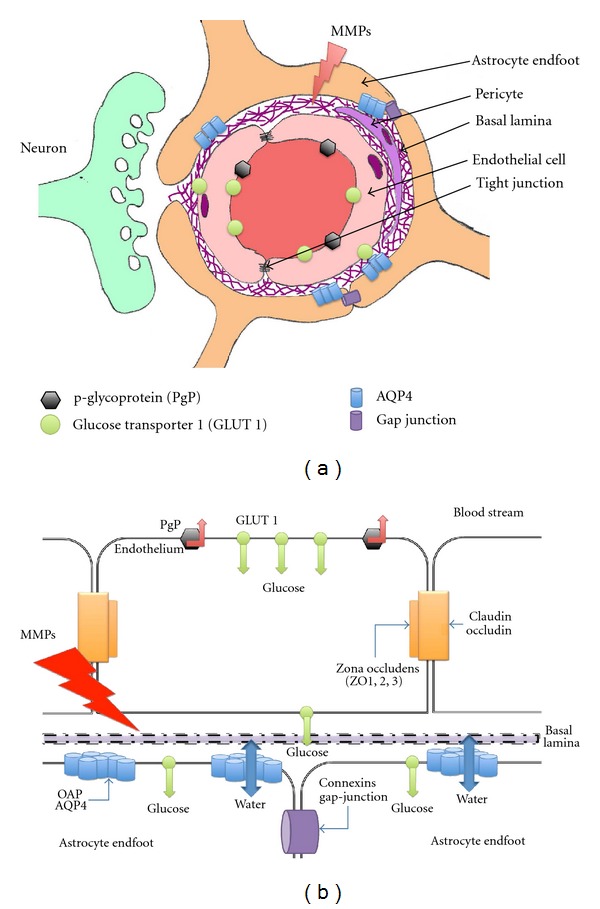
(a) Schematic drawing of the neurovascular unit (NVU) in the capillary bed composed by the neuron, astrocyte endfoot, basal lamina, pericyte, and endothelial cell. The endothelial cell is the first barrier between the blood stream and the nervous tissue. The presence of the tight junction composes the physical barrier and the movement of substrates is controlled by several transporters. The astrocyte endfeet are linked with the gap junction, allowing movement of several solutes in the astrocyte network. The basal lamina is composed of several proteins such as agrin, dystroglycan, and perlecan. (b) A close-up schematic drawing of the endothelial cells and astrocyte endfeet with some of the proteins involved in edema formation and resolution.

The BBB is located in the endothelial cells of brain vessels, with the presence of tight junctions and adherens junctions between the cells (Figure 1) that prevent paracellular diffusion and act as a unit to regulate ions and other molecules between peripheral blood flow and brain parenchyma. Tight junctions are composed of several protein families: trans-membrane proteins (claudins and occludins), cytoplasmic proteins, and zona occludens proteins. They bind the afore mentioned proteins with structural cytoskeletal proteins such as actin. Adherens junctions are formed by proteins such as platelet-endothelial cell adhesion molecule (PECAM) and vascular endothelial-cadherin, which contribute to the close physical contact between endothelial cells and facilitate the formation of tight junctions.

 The brain endothelial cells of the BBB also present specific transport proteins located on the luminal and abluminal membranes for nutrients, ions, and toxins to cross the endothelial layer between the blood stream and brain [13, 16]. For example, energy molecules are transported by specific solute carriers such as glucose transporter 1 (GLUT 1) and monocarboxylate transporters 1 and 2 (MCT1, MCT2). Large molecular weight solutes (e.g., large proteins and peptides) are able to cross the BBB and enter the intact CNS via endocytotic mechanisms called receptor-mediated transcytosis, such as with insulin, or adsorptive-mediated transcytosis, exemplified by albumin. On the other hand, transport can also be achieved by the ATP-binding protein (ABC) family, which consumes ATP to effectively transport a wide range of lipid-soluble compounds from the brain endothelium. In the BBB examples of ABC transporters for efflux transport are P-glycoprotein (P-gp), multidrug resistance-associated protein (MRP), and breast cancer resistance protein (BCRP) [16]. These efflux transporters are understood as gatekeepers of the brain because they keep tight control over which substances are allowed to enter the CNS through the endothelial cell barrier (Figure 1). Endothelial cells also present a metabolic barrier of the BBB, which functions to inactivate molecules capable of penetrating cerebral endothelial cells.

Quite recently it has been proposed that the primary barrier of the BBB may extend to the basal lamina, thus preventing the entry of immune cells into the parenchyma under normal brain conditions [12]. Historically the brain was thought to be an immune cell deficient organ, and the BBB was thought to prevent passage of any immune cells into the brain. However, peripheral immune cells from the blood have been observed to enter and be present in the brain at multiple time points during embryonic development [17] and in normal physiological conditions in adults [12]. Therefore, the theory of the CNS as an immune-independent organ has recently started to be reexamined and revised. Engelhardt and collaborators elegantly compare the perivascular space as a castle moat with perivascular antigen presenting cells floating as guards, confined by the inner and outer wall, which is the basement membrane of the astrocytic endfeet and the endothelial cell, respectively [12]. Endothelial cells and other cells, such as the astrocytes, may also contribute to the tight regulation of the movement of immune cells between the peripheral blood stream and the brain. However, the exact mechanisms by which peripheral cells enter the brain are still a matter of discussion. Moreover, rather than the BBB being a rigid wall, it provides a dynamic interface between the brain and the rest of the body.

As mentioned previously, the presence and the maintenance of these barrier properties are important for brain homeostasis and for neuronal functioning [13]. In fact, disruption of tight junctions leads to BBB disruption and extravasation of blood components and water, which contribute to vasogenic edema formation. We will cover these in more detail in the following section.

## 3. Edema Process after Stroke: Endothelium and Astrocyte, *Concerto en Duo*

### 3.1. BBB Disruption and Edema Formation

Cerebral edema has been traditionally divided into 2 major classes: cytotoxic and vasogenic [18] for cerebrovascular diseases and other brain pathologies. Cytotoxic edema is defined by intracellular accumulation of water coming from the extracellular space without BBB disruption. Vasogenic edema appears after BBB disruption, leading to a diffusion of proteins from the blood to the tissue followed by water accumulation in the extracellular space [18]. However, this division alone does not explain fully the diversity and the complexity of the edema process in brain ischemia as well as in the other brain injuries and disorders. Based on several recent advances in the understanding of the molecular mechanisms of edema formation and BBB properties, a third subtype of edematous processes was named *ionic edema* and described as a continuum between the cytotoxic to vasogenic edema in the cerebrovascular diseases [19, 20]. In fact, cytotoxic, or anoxic, edema occurs within the first few minutes after cerebral blood flow stoppage and is characterized as swelling of the astrocytes and neuronal dendrites [20, 21]. The cellular swelling within the first 10 minutes is a result of oxygen and glucose deprivation followed by a slow rise in extracellular [K^+^] [22]. The absence of oxygen and energy nutrients induces a disruption of the cellular ionic gradients and leads to entry of ions into cells. Water follows this ionic gradient into the cells and induces cellular swelling. Cytotoxic/anoxic edema may evolve quickly to become ionic edema because the absence of oxygen and nutrients further alters the energy balance in endothelial cells and the ionic gradients, including transcapillary flux of Na^+^ in these cells [19, 23]. The endothelial cells also require a large amount of ATP production, characterized by the high density of mitochondria, which are important for the regular homeostatic BBB functions such as maintenance of ionic gradients and membrane transporters [24, 25]. The absence of energy supplies for these cells would severely impair these functions. Reperfusion induces overpressure accompanied by shear stress on the nonperfused vascular tree that results in early transient leakage of the BBB [26, 27]. This leakage results in further entry of water through the endothelial cells resulting in brain swelling within 30 minutes after reperfusion [26, 27] and additional BBB permeability [27, 28]. This early opening of the BBB has also been described clinically in humans and is frequently associated with hemorrhagic transformation [29]. Early reperfusion probably mitigates the BBB alterations, but if it is delayed, reperfusion will exacerbate the amount of endothelial injury [30–32]. The final step is the development of vasogenic edema, in which there is disruption of cerebrovascular endothelial tight junctions leading to increased permeability to albumin and other plasma proteins [18]. Another contributing factor of brain edema formation in addition to tight junction disruption is brain endothelial transcytosis [33]. BBB disruption is usually coupled with the inflammatory response and activation of matrix metalloproteinases (MMP) [34, 35]. In fact, vasogenic edema development is aggravated by MMP-9, which degrades basal lamina, the connection between astrocytic endfeet and endothelial cells [36]. 

In the clinic, diffusion-weighted imaging (DWI) and T2-weighted imaging (T2WI) magnetic resonance imaging (MRI) modalities are used extensively to assess postischemic edema [20, 37, 38]. T2 values represent water content and apparent diffusion coefficient (ADC) values derived from DWI images represent water mobility in the tissue [20, 37]. ADC values decrease rapidly after stroke onset, indicating restricting water movement, and are interpreted as evidence of ionic edema with the characteristic swelling of the brain cells causing a decrease in extracellular space as proposed in our classification mentioned before. T2 values increase at later time points, which are associated with vasogenic edema [20, 39]. 

The molecular mechanisms and temporal development of edema after stroke have been well studied. However, the cellular and molecular mechanisms involved in edema resolution are not well understood in stroke and other brain diseases. The healing of the endothelial cells with stabilization of the tight junctions may be a critical step to limit the entry of blood components into the brain. Thus, stabilizing the NVU may be an essential component of controlling edema formation and BBB breakdown after stroke.

Postischemic BBB disruption has been commonly believed to be biphasic [40], but recent work suggests that the BBB disruption may be continuous for up to 5 weeks after ischemia in rats [28]. BBB leakage was demonstrated using gadolinium and magnetic resonance imaging (MRI) at 25 min; 2, 4, 6, 12, 18, 24, 36, 48, and 72 hours; and 1, 2, 3, 4, and 5 weeks after ischemia [28]. Similarly, albumin leakage through the BBB, especially in the hippocampus, has also been observed in spontaneously hypertensive stroke prone rats long term [41]. Although these data do not completely rule out the possibility of a biphasic pattern in the opening of the BBB, the long-term leakage of the BBB is important to note from the standpoint of postischemic edema because this disruption could account for a prolonged vasogenic edema. 

### 3.2. “*Concerto en Duo*”: Astrocyte Network in Edema Formation and Resolution

As part of the NVU the astrocyte endfeet in contact to the blood vessels are well known for to swell after stroke [42–44]. The recent knowledge on the transporters and channels in this astrocyte subdomain gives new perspectives on the understanding of astrocyte swelling. In fact, aquaporin 4 (AQP4), a member of the family of 13 water channel proteins, is proposed to have an important role in edema formation [20, 45]. AQP4 is the most abundant water channel in the brain, in part due to its high concentration on astrocytic endfeet which are in contact with all the cerebral blood vessels [46, 47]. More recently, AQP1 has also been described in a subpopulation of astrocytes within the nonhuman primate but not in rodents, suggesting interspecies differences and a possible role in brain water homeostasis [48]. AQP1 has also been reported to be present in peripheral endothelia and primary rat brain endothelial cell cultures [49]. Interestingly, Dolman and collaborators observed that mRNA AQP1 levels were lower in cultured brain endothelial cells when cocultured with astrocytes [49], suggesting an inhibition effect of the astrocytes on the AQP expression in endothelia. In fact, there are publications reporting a low level of AQP in endothelial cells *in vivo* [50], although AQP is more abundant in astrocytes [49, 51–54]. 

**Figure 2:**
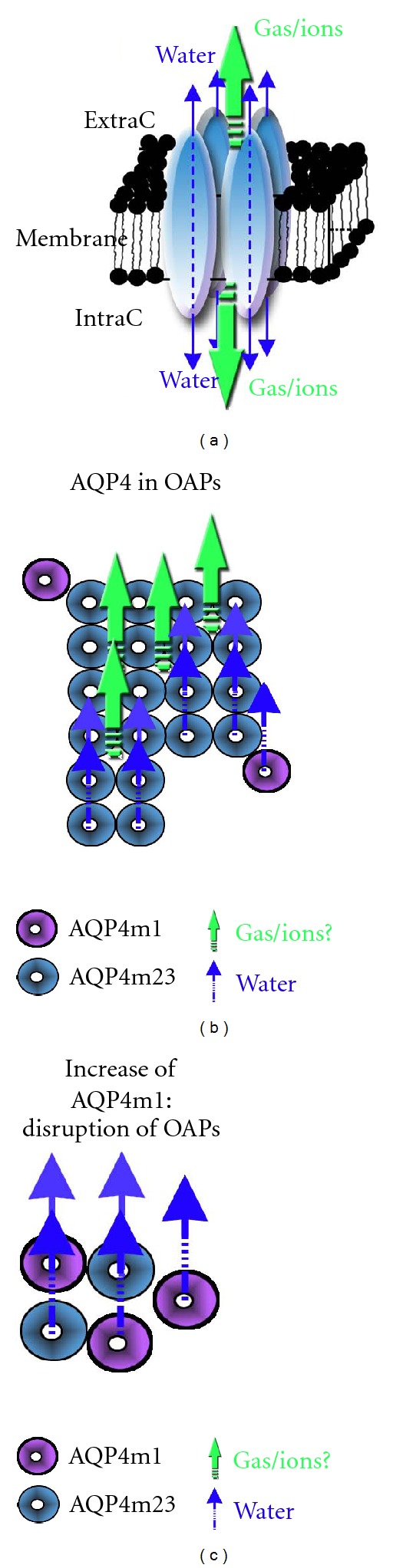
(a) Schematic drawing of the aquaporin homotetramer assembly within the lipid membrane: the central pore is proposed to be permeable to cations and gases (green arrows). Each individual aquaporin facilitates bidirectional water movement depending on the osmotic gradient (blue arrows). (b) AQP4 homotetramer is assembled in a higher structure named orthogonal array of particles (OAPs). Two isoforms of AQP4, AQP4-M1 (purple circles) and AQP4-M23 (blue circles) isoforms, contribute to the formation of OAPs. *In vitro* experiment showed that higher expression of AQP4-M23 contributes to the formation of larger OAPs. (c) Increase of AQP4-M1 induced disruption of OAPs. Recent knowledge on AQP leads us to hypothesize that the large OAPs contribute to gas and cation diffusion in the astrocyte membranes through central pores (green arrows).

Currently, AQP4 is considered as a key player in the edema process by its location on the astrocyte endfeet [20, 55]. AQP4 is assembled in homotetramers where each individual aquaporin represents a water channel [56]. Interestingly, AQP4 is also organized in the astrocyte endfeet membrane in a larger geometric structure known as an orthogonal array of particles (OAPs), which has been described with freeze-fracture techniques and electron microscopy studies (Figure 2) [57]. OAPs are present in all astrocyte endfeet in contact with the blood vessels as well as the glia limitans. OAPs are formed with two isoforms of AQP4: long (AQP4-M1) and short splice variants (AQP4-M23). The ratio of AQP4-M1 to AQP4-M23 determines the size of these OAPs [57] in contact with the basal lamina of brain vessels (Figure 1). Experiments in oocytes showed that the AQP4-M23 isoform stabilizes the OAP structure [57, 58]. However, the exact functional roles of the OAPs remain unknown in normal and pathological conditions. Recently, AQP4-m1 mRNA and protein were found to increase quickly after stroke onset, while AQP4-m23 remained the same. The increase of AQP4-m1 early after ischemia could favor a shift toward M1 in the M1/M23 balance, which is known to favor small size OAPs [27]. In accordance with this work, previous studies have shown that early disorganization of OAPs on the astrocyte endfeet after global cerebral ischemia preceded astrocyte swelling [59]. Although a direct effect of the modification in the ratio of AQP4-M1 to AQP4-M23 on water permeability has not yet been directly investigated *in vivo*, in a preconditioning model, a strong increase in AQP4 expression and increase of AQP4-M1 were correlated with reduced edema and less water in the tissue, suggesting increased water diffusibility which resulted in the removal of excess liquid from the brain tissue [27]. Interestingly, it was recently proposed that the assemblage of 4 aquaporin molecules forms a central pore, through which water, ions, and gases may flow depending on the AQP subtype. For example, the central pore is permeable for O_2_, CO_2_, and possibly nitric oxide for AQP1, 4, and 5 [56, 60]. Thus, the disruption of the OAPs may also affect the diffusion of ions and gas through the central pore.

 Due to its location in the astrocyte endfeet in contact with the blood vessels, AQP4 has been proposed to be linked with BBB integrity [52, 61, 62] and cell adhesion [63]. In the epithelial cells of the eye lens AQP0 is present in the OAPs and participates in epithelial cells linkage; however it does not facilitate water flux [64]. In this case, the presence of AQP4 in the astrocyte endfeet membrane was dependent on the presence of proteins in the basal lamina such as agrin, *α*-dystroglycan, and laminin [65, 66] in addition to syntrophin and dystrophin protein complexes [67, 68]. The connection of AQP4 to proteins in the basal lamina may explain the ability of astrocytes to maintain the integrity of the blood-brain barrier, suggesting a possible role for AQP4 as a structural molecule within the perivascular space [61]. However, reports using AQP4 knock out (AQP4-KO) mice show contradicting results regarding the modifications in the BBB structure suggesting that AQP4 may not be integral to the BBB structure [61, 69]. Similarly in our siRNA silencing studies, BBB permeability was not significantly changed at distance from the site of injection after injection of siRNA against AQP4, even though AQP4 expression was decreased [55]. We also showed that the upregulation of AQP4 in a preconditioning model did not prevent the early opening of the BBB after stroke [27].

Heparan sulfate proteoglycan is a large family of proteins with agrin and perlecan, involved in the basal lamina composition located between the astrocyte endfeet and endothelial cells [54, 70]. Agrin and dystroglycan seem to play an integral role in the maintenance of astrocyte polarity by the interaction with AQP4 in the astrocyte endfeet [54]. Specifically, agrin KO mice showed a significantly decreased density of OAP in the astrocyte endfeet when compared to wildype but overall immunoreactivity of AQP4 did not differ significantly [71]. Dysfunctions in the basal lamina are related to increase of the BBB disruption, promoting edema formation. In fact, a family of endopeptidases, matrix metalloproteinases (MMPs), has been shown to degrade the proteins of the basal lamina and contribute to vasogenic cerebral edema [36]. In the human brain, MMPs are usually very low in concentration under nonpathological conditions [72]. However, after injuries such as ischemic stroke, certain MMPs such as MMP-2, -3, and -7 and especially MMP-9 have been shown to be upregulated in the brain (reviewed in [72]). This layer between astrocytes and endothelial cells is a potential future target for the NVU protection. Recently, Dr. Bix and collaborators have shown that administration of perlecan domain V, which is the c-terminal fragment, administered 24 hours after ischemic stroke has beneficial effects by interacting with integrins [73]. Perlecan domain V increased expression of vascular endothelial growth factor (VEGF), thus promoting angiogenesis, and interestingly did not lead to increased BBB permeability [73] even though VEGF is known to increase BBB permeability after ischemia [74]. Perlecan has also been shown to modulate postischemic astrogliosis through interaction with dystroglycans and integrins in the astrocytes [75]. 

Astrocytic AQP4 is not only linked with the matrix proteins but also with several other channels present in higher concentration in the astrocyte endfeet such as potassium inner rectifying channel 4.1 (KIR4.1), connexins (Cx), and also chloride channel 2 (CIC-2) [76, 77]. Colocalization of AQP4 and KIR4.1 suggests that AQP4 may have a role in potassium homeostasis by facilitating water diffusion along the potassium gradient and AQP4-KO mice display a delay in potassium reuptake during electrical activity [76]. The decrease of AQP4 expression using siRNA showed an associative decrease of connexin 43 (Cx43), a protein involved in gap junction formation, and a decrease of CIC-2, involved in the regulatory volume decrease function of the astrocytes. Interestingly, gap junctions and AQP4 are morphologically closely associated [78] with the astrocyte endfeet. The gap junctions in the astrocyte contribute to the formation of a complex network named the astroglial network [79]. Intercellular and intracellular communication that facilitate the movement of second messengers, amino acids, nucleotides, energy metabolites, and small peptides [79–82] in astrocyte processes occur through gap junctions, which are made up of a family of channel proteins called connexins [83, 84]. In astrocytes, Cx30 and Cx43 are predominant [83–85]. However, it is also important to note that Cx43, along with Cx37, Cx40 [86, 87], and Cx45 [87], is also expressed in brain endothelial cells. The protein level of Cx40 and Cx45 was shown to increase in cerebral arteries, but no change in protein or mRNA was observed for brain endothelial Cx43 and Cx37 after a model of brain injury causing cerebral vascular dysfunction [87]. The effect of astrocytic Cx43 upregulation or downregulation after ischemia still remains controversial and there is no consensus as to what provides beneficial effects [88]. However, in humans, there are reports that show that Cx43 protein levels were increased in the penumbra [89]. And because Cx43 and Cx30 knockouts have been observed to be more edema prone [90], it is possible that the increase in Cx43 after ischemia may be a physiological response to decrease edema. The induction of Cx43 may be facilitating water flow throughout the astrocyte network to diversify and dissipate the accumulation of fluid from just one region. From these data we hypothesize that gap junction proteins, specifically Cx43 on astrocytes, are working with AQP4. Evidence for this also comes from a significant decrease of Cx43 observed in mouse astrocyte cell cultures after administration of small interference RNA against AQP4 [91]. Although direct functional data are still lacking, one possibility is that AQP4 and Cx43 is working together to direct water flow between astrocytes and could be controlling astrocytic swelling. 

The role of AQP4 in cerebral edema formation and resolution has been studied in several models. However the precise role of AQP4 remains unclear and depends on the pathological model used [92, 93]. Indeed, the absence of AQP4 was shown to prevent the formation of edema in a permanent ischemia model in AQP4-KO mice [94]. Similarly, edema formation is prevented in *α*-syntrophin knockout mice at 24 h after stroke [68]. This decrease of brain swelling was correlated with the loss of the perivascular AQP4 domain in *α*-syntrophin-KO mice [68]. These results suggest that perivascular AQP4 has an important role in edema formation. However, the absence of AQP4 in AQP4-KO mice also prevents water clearance in an experiment of intrastriatal infusion of a saline solution, showing that AQP4 is critical for water removal from tissue [95]. Conversely, in a preconditioning stroke model, a higher induction of AQP4 was correlated with edema reduction [27]. However, this reduction of edema may be referring to vasogenic edema, in which case, AQP4 is said to aid in edema resolution by actively pumping out water from the cerebral tissue to peripheral blood [95]. The redistribution of the water in the astrocyte compartment through the astrocyte network would also be possible for the CSF compartments. This hypothesis is supported by a publication showing an increase of AQP4 in ependymal cells in the border of the ventricles in a traumatic brain injury model [96].

To summarize, the exact mechanism causing decreased edema formation is not yet fully understood, but AQP4 and the astrocyte network with the gap-junction proteins may certainly be contributing. Osmotic gradients can also play an important role, and recently, high AQP4 expression was observed in hypersaline treatment after stroke correlating with decreased edema formation at 48 hours [97].

## 4. rtPA: A Unique Drug for Stroke Treatment with Aversive Effects on the NVU

### 4.1. Clinical Evidence (from Bed to the Bench, Neurotoxicity of rtPA)

As discussed in the introduction, recombinant tissue plasminogen activator (rtPA) is currently the only thrombolytic molecule FDA approved for treatment of acute ischemic stroke [4]. The intact BBB is usually an obstacle for most neuropharmacological agents in healthy patients. The dysfunction of the BBB after ischemia could cause problems for the therapeutic function of rtPA. This protease targets fibrin-bound plasminogens and converts them into plasmins, which then cut the fibrin clot and lyse it. Intravenously infused at a dose of 0.9 mg/kg over one hour, rtPA provides increased survival and better neurological outcomes [4]. To be beneficial for the patient, rtPA must be administered within the first 4 h 30 min after stroke onset [5, 6]. Despite the organization of emergency care, only 5% of stroke patients are eligible for this therapy. In fact, late administration of rtPA translated to a higher risk of bleeding and extension of the lesion [7]. Higher doses of rtPA do not bind only the fibrin clot but also activate the circulating plasminogen activator (tPA). This activation contributes to a generalized fibrinolysis and fibrinogenolysis, which is suspected to be a cause of bleeding. But the mechanisms of the hemorrhagic transformation after rtPA treatment seem to be more complex than can be accounted for by the affinity of rtPA for fibrin alone. In fact, the enhanced fibrin specificity of tenecteplase and reteplase, two rtPA derivatives, resulted in no significant difference in terms of cerebral hemorrhage [98, 99]. 

Interestingly, the comparison with myocardial infarction shows a low incidence of cerebral hemorrhage after rtPA administration [100] suggesting a direct link between bleeding and the ischemic pathophysiology. Clinical studies showed that 80% of bleeding after cerebral thrombolysis occur preferentially in the ischemic territory [7].

### 4.2. Aversive Effects of rtPA Treatment on the NVU after Stroke

To have a better understanding of the aversive effects of rtPA its neurotoxic effects were examined. It is well known that endogenous tPA is present in the blood stream, endothelial cells, neurons, and microglial cells [101]. In the brain parenchyma, tPA activity was found to be pleiotropic and associated with synaptic plasticity and cell death [102–104]. In fact, tPA interacts with several neuronal proteins such as N-methyl-D-aspartate (NMDA) receptors, one subtype of glutamatergic receptors, low-density lipoprotein-receptor-related protein (LRP), and Annexin-II [101, 105, 106]. tPA is synthesized in neurons, stored in presynaptic vesicles, and released following depolarization in synergy with the neurotransmitters. In the synaptic cleft, tPA binds and cleaves the NR1 subunit of NMDA receptors that causes an amplification of calcium influx in postsynaptic neurons and an increase of the glutamatergic response in physiological conditions. However, this physiological response becomes excitotoxic after ischemia and is magnified after rtPA injection [101, 107, 108]. The injection of antibodies against the NR1-subunit prevented these proexcitotoxic effects of endogenous tPA and reduced brain infarction and BBB leakage after stroke [109]. These data suggest that the NMDA receptor may be a protective drug target for the NVU after stroke and may provide a potential extension of the rtPA therapeutic window [109]. 

The presence of rtPA in the brain parenchyma has been explained by its passage through the BBB in several *in vitro* models with different proposed mechanisms.

- rtPA diffuses into the brain parenchyma through an already opened BBB as a consequence of the ischemic process. As we discussed previously, the kinetics of the BBB opening is complex in the early stages after stroke and it is difficult to observe this with clinical imaging [29]. Interestingly, *in vitro* endothelial monolayer cultured with astrocytes enables us to observe the ability of rtPA to cross the intact BBB [110], which is increased under oxygen-glucose deprivation (OGD) [111]. Therefore, as rtPA potentially diffuses through an open or closed BBB in early time points after stroke onset, it may aggravate neuronal cell death as described previously.
- rtPA could cross the BBB by degrading the endothelium via its own proteolytic activity, but it is not a requirement in the intact BBB [110]. The ability of rtPA to cross the intact BBB at a thrombolytic dose suggests that this protease may interact first with the endothelial cells before the BBB breakdown. In fact, rtPA promotes breakdown of the BBB [112] by stimulating the synthesis activity of MMP-9 [113–116] and other MMP isoforms [117] exacerbating the degradation of the basal lamina and subsequent vasogenic edema formation and hemorrhage. The thrombolytic products could exacerbate the proposed mechanism [118].
- Finally, LRP potentially contributes in trans-endothelial transport of the exogenous rtPA [106, 119, 120] and then activates the astrocytic MMP-9 and nuclear factor NF-*κ*B, which promotes the expression of inducible nitric oxide synthase (iNOS). This increase of NO results in increased BBB permeability [121].

With all these data together, Yepes and collaborators have proposed the following potential cellular and molecular events to explain the toxicity of the rtPA and tPA on the NVU [104].

1. Circulating endogenous tPA and rtPA cross the BBB (intact or damaged endothelial layer) and increase MMP-9 activity in the basal lamina soon after stroke onset which compromises the NVU integrity and makes it fragile.
2. Then tPA and rtPA bind to the astrocytic LRP, inducing the loss of the extracellular domain of LRP [122, 123] in the basal lamina, and release the intracellular domain of LRP in the astrocytic cytoplasm to activate NF-*κ*B. This NF-*κ*B activation increases iNOS and MMP9 expression and overall function in the whole NVU, causing separation of astrocytic endfeet from the basal lamina. This is usually observed at the later stages of BBB breakdown. However, it is tempting to speculate that this cascade, which involves the perivascular cells of the NVU, would be an accelerated pathological process resulting from the use of rtPA. It is possible that rtPA and tPA may also affect the phenotype of the astrocyte endfeet by changes in the level of expression of key proteins such as AQP4 and also Cx43.

### 4.3. New Therapeutic Strategies for rtPA Treatment after Stroke

The BBB is definitely not a barrier to rtPA in stroke but the BBB does become a serious barrier to the effective usage of this drug in clinic due to the neurotoxic effects and the risk of hemorrhagic transformation. Interestingly, tPA may be endogenously synthesized by the central nervous system in neurons and endothelial cells [124]. However, tPA and rtPA have effects on the endothelial cells, astrocytes, and neurons and possibly other glial cell types such as oligodendrocytes and microglia. In order to prevent the aversive effects of rtPA while maintaining the benefits of early reperfusion, several new therapeutic strategies have been examined to prevent the interaction of rtPA with the NMDA receptor within the NVU [104]. In fact, NMDA receptors are expressed not only in neurons but also in oligodendrocytes and endothelial cells [125, 126]. One of these strategies uses an LRP antagonist (RAP) to minimize the binding of rtPA with LRP in the endothelial cells. A second strategy uses the ATD-NR1 antibody to block rtPA binding of the NR1 subunit on neuronal NMDA receptors. The last one uses a mutation of the rtPA to decrease its adverse effects on the nervous tissue [104]. An example of a natural drug, desmoteplase, the vampire bat *Desmodus Rotundus* Salivary Plasminogen Activator (DSPA), is a thrombolytic agent under development. It shows little neurotoxicity and has the ability to interact with the BBB endothelium through the same receptor (LRP) as that of tPA [127, 128]. Unfortunately, the clinical trial of DIAS-2 (Desmoteplase In Acute ischemic Stroke) showed no benefit of the desmoteplase versus placebo [129]. Although the outcome of this clinical trial was disappointing, promising alternatives pathways are being investigated. In fact, Gleevec, a FDA approved drug for treatment of chronic myelogenous leukemia, was recently proposed to prevent the complications associated with rtPA treatment [130]. Gleevec inhibits the activation of platelet-derived growth factor alpha receptor (PDGFR). It was shown that tPA increases BBB permeability through the indirect activation of perivascular astrocytic PDGFR [130]. 

MMP inhibition is a good strategy based on reports of easy monitoring of MMP blood levels, defining them as potential biomarkers of brain damage [131, 132]. But because endogenous MMPs are also key mediators in stroke recovery by contributing to inflammatory and remodeling responses, pharmacological targeting must be accurately applied for acute stroke phases so; their beneficial effects are not compromised [133, 134]. Despite efforts to understand the complex link between BBB integrity and the hemorrhage risk [112], a better definition and understanding of NVU kinetics and the mechanisms underlying their dysfunction is still needed to better define eligibility criteria for rtPA treatment. Thus, alternative approaches other than MMP inhibition as mentioned before in some recent developments will offer interesting treatment strategies after stroke.

## 5. NVU Protection May Be the Future instead of Neuroprotection in Stroke Treatment 

### 5.1. Preconditioning for Future Development of New Drugs

Given the small number of patients eligible for thrombolysis, many pharmaceutical compounds have been developed to limit the progression of brain injury by targeting different mechanisms leading to neuronal death [135]. Despite promising protective effects observed in preclinical studies, no compound to date has demonstrated benefit against stroke-induced neuronal death after facing the rigorous wall of clinical trials [136]. 

As mentioned in Section 1, research on brain diseases has focused on neuronal damage, as it was thought to be the major cause of cognitive deficits. However, ischemic stroke is a complex brain disease characterized by sudden onset of disabilities related to brain damage with a vascular origin. Because the development of many neuroprotective molecules for treatment over the last twenty years has been unsuccessful, researchers have switched gears towards investigating the natural endogenous neuroprotection of ischemic tolerance [137]. The purpose of the ischemic tolerance preconditioning is to induce endogenous defense mechanisms prior to the ischemic event that will attenuate the eventual consequences of ischemia. This resistance to ischemic damage can be achieved experimentally by several stimuli including ischemic preconditioning [138]. The concept and protocols were adapted from previous studies done in myocardial infarction. In fact, a short duration of coronary occlusion is unable to cause myocyte necrosis. However, when carried out before a prolonged occlusion, a short occlusion significantly reduced the final infarct volume of the myocardium [139]. This initial nonharmful ischemic insult triggered endogenous mechanisms that made the organ more resistant to the next attack for up to two periods of ischemic tolerance [139]. The first period of ischemic tolerance resulted from posttranscriptional responses and began minutes after preconditioning. The second, longer period, began 24 hours after preconditioning and lasted up to 7 days with maximal protection found at 3 days. 

As with the cardiac preconditioning, ischemic tolerance in the brain also has delayed mechanisms leading to neuroprotection [140]. However, the mechanisms are complex and not well understood. The induction of ischemic tolerance likely depends on the coordinated responses at the genomic, molecular, cellular, and tissue levels [141–143], which suggests the importance of the interactions between the astrocyte and endothelial cells in the NVU. Regarding neurovascular events in stroke pathophysiology, there has been a growing interest in vascular approaches to the preconditioning mechanisms. Protective effects of preconditioning were observed *in vivo*, demonstrating that endothelium function is preserved by improving cerebral blood flow during reperfusion in areas surrounding the lesion [144], and that BBB integrity is maintained with a reduction in edema formation [145]. The induced protection was again correlated not only with a decreased expression of MMP-9 [146] but also with a reduced neutrophil adhesion to endothelial cells through a decreased expression of ICAM-1 [147, 148]. These results were confirmed by *in vitro* studies that report a protective effect via preservation of BBB integrity, by both a decreased expression of the inflammatory molecules ICAM-1 and VCAM-1 [149, 150] and maintenance of tight junction structure [149]. Moreover, preconditioning also facilitates the increase of AQP4 expression at early time-points after stroke onset, which is associated with a decrease of the edema formation [27]. A recent study also reported the protective role of glial tissue preconditioning in severe stroke [151]. These recent observations suggest that future drug development must focus on drugs affecting the entire NVU instead of one cell type as was proposed in the 1990s with the development of calcium channel and NMDA inhibitors. Recently, some compounds like edaravone, an antioxidant, showed benefits in preclinical and clinical studies by protection of the NVU [152, 153]. But further trials are needed to confirm these promising preliminary results [154].

### 5.2. Protection of the NVU: Focus on PPARs

Preventive neuroprotection also involves management of risk factors, which is supported by studies showing that physical exercise [155] or lipid-lowering treatment reduces the occurrence and severity of stroke [156–158]. In this context, the involvement of pharmacological agents that are activators of nuclear receptors like peroxisome proliferator-activated receptors (PPARs) could be a promising study. Present in three isoforms, *α*, *β*/*δ*, and *γ*, these receptors exhibit pleiotropic activity in the sense that they can activate or repress the transcription of many genes involved in lipid and carbohydrate metabolism in addition to inflammation [159, 160]. PPARs are expressed in neurons, endothelial cells, and glial cells [161]. Activation of the PPARs has long-term effects lasting from hours to days, which correspond to an activation of gene transcription (named transactivation) as has been seen in lipid and carbohydrate metabolism. However, activation of the PPARs induce a cellular response within minutes to hours and this corresponds to an inhibition of gene transcription named transrepression [162]. The latter mechanism does not require binding to DNA, but rather protein-protein interaction involving other transcription factors like NF-*κ*B of STAT-3 and AP-1, to inhibit their activity as reported for inflammatory genes [163].

Independent of its lipid-lowering activity, PPAR-*α* activation was found to be neuroprotective in several *in vivo* studies carried out in mice subjected to transient ischemia with preventive or curative treatments by agonists such as fenofibrate, WY-14643, and resveratrol (a polyphenol present in grapes) [164–166]. The observed protection is the result of an anti-inflammatory mechanism, which decreases the expression of adhesion molecules, ICAM-1 and VCAM-1, in brain endothelial cells. Effects of antioxidants were also observed. However, a study using a BBB *in vitro* model combining endothelial cells with glial cells from wild-type or PPAR-*α* knockout mice has demonstrated not only that the observed protection against OGD-induced hyperpermeability was dependent on this nuclear receptor activation but also that the ligand targeted specifically the endothelial cells without modulation of the classical PPAR-*α* target genes associated with inflammation or metabolism [167]. Moreover, protective effects of PPAR-*γ* were not only reported through similar mechanisms [168] but also via an inhibition of NF*κ*B and TNF-*α* pathways [169, 170] and macrophages/microglial cells activation, thus preventing cytokine production [171]. One study also suggests that PPAR-*γ* agonists could inhibit excitotoxicity-induced neuronal death [172]. 

Statins are HMG-CoA reductase inhibitors. This enzyme catalyzes the conversion of HMG-CoA (3-hydroxy-3-methylglutaryl coenzyme A) to mevalonate, a precursor of cholesterol. As lipid lowering agents statins also exert pleiotropic effects at the vascular level [173]. In addition to protection against excitotoxicity in cultured neurons [174], statins have demonstrated preservation of BBB endothelial cells' integrity against glutamate excitotoxic challenge *in vitro* [175]. These compounds also enabled the reduction of MMP-9 synthesis in rtPA-activated astrocytes [176]. The effects of statins may involve nuclear receptors, through an increase in both expression and activity of PPAR-*α* [177–179]. More recently, brain endothelial PPAR-*δ* activation has proven to be protective against ischemia-induced cell death through inhibition of the miR-15a microRNA, thus strengthening the therapeutic concept based on activation of PPARs for the treatment of stroke-related microvascular dysfunction [180].

### 5.3. Inhibition of JNK Activation and NVU Protection

The c-Jun N-terminal kinases (JNKs) belong to the mitogen activated protein kinase (MAPK) family; the two other members being p38 and ERK [181, 182]. The isoforms JNK1 and JNK2 are ubiquitously distributed, while JNK3 is primarily expressed in the heart, brain, endocrine pancreas and testis [183]. JNKs are activated by phosphorylation, which is catalyzed by upstream kinases—MKK 4 and 7 [182–184]. JNK activation is essential for normal brain development and organogenesis during embryonic development [185]. However, the activation of JNKs plays several roles ranging from regulation of cell survival and apoptosis to cell proliferation [183, 185–187]. They are activated under pathological conditions both in the brain [188, 189] and in the periphery [190, 191]. In fact, JNK phosphorylation initially decreases after stroke and then starts to increase at 1.5 hours with a maximum at 9 hours after onset [192]. Phosphorylation of c-Jun, a JNK substrate, follows the same temporal pattern, peaking at 8 hours post-stroke [192, 193]. 

The development of the peptide named DJNKi, a competitive inhibitor of the JNK signaling pathway, has been shown to reduce lesion volume of mice with transient MCAO by 90% even when induced 6 hours after injury. This lesion volume decrease was accompanied by behavior improvements as well [193], suggesting an increase of the therapeutic time window almost 2 times longer than tPA. This positive outcome was also observed in a more severe model with a permanent occlusion model [194]. Moreover, DJNKi has been shown to be compatible for treatment of ischemic stroke even in the presence of rtPA and was shown to decrease lesion volume [195]. DJNKi also improved neurobehavior scores and decreased hemispheric swelling after a model of intra-cerebral hemorrhage [196]. Thus, DJNKi could possibly attenuate the highly probable side effect of hemorrhagic transformation caused by rtPA. Interestingly, in this model of intracerebral hemorrhage, DJNKi administration significantly increased AQP4 expression 48 hours after injury. This increase in AQP4 expression negatively correlated with decreased hemispheric swelling, thus pointing towards a possible role of DJNKi controlling edema as well. In fact, activation of the JNK pathway is present not only in the neurons but also in glial cells [197] and brain endothelial cells [198]. Such activation in nonneuronal cells may negatively impact neuronal cell death and function [197]. In the context of broad effects of this drug, Benakis et al. [199] showed that DJNKI-1, injected peripherally, is able to modulate some nonneuronal inflammatory processes. As discussed previously, the development of a drug targeting several cells such as in the NVU may help to move towards success in the clinic.

## 6. Summary and Perspectives in ****Stroke Research

In summary, the data found in the literature suggest that the failure of agents in protecting the brain against stroke may come from the fact that each developed compound targeted only one mechanism and one cell type of stroke pathophysiology. Ischemic preconditioning appears to be an attractive experimental strategy that would identify endogenous mechanisms of protection and regeneration. Recent evidence of such protective mechanisms supports a complex action on cells of the NVU, underlining the importance of the interactions between endothelial cells and astrocytes in the pathophysiology after stroke. As our knowledge of the NVU increases, molecules with pleiotropic activity will become increasing useful in the development of post-ischemic treatments in the clinics.
